# Effect of pasteurized *Akkermansia muciniphila* MucT on insulin sensitivity, body composition, and GLP-1 production in subjects with metabolic syndrome: impact of low baseline gut *Akkermansia* levels

**DOI:** 10.1080/19490976.2026.2690689

**Published:** 2026-06-24

**Authors:** Peter Suenaert, Anneleen Segers, Leen Rymenans, Hélène Devroye, Janne Marie Moll, Patrice D. Cani, Willem M. de Vos

**Affiliations:** a Research and Development, The Akkermansia Company, Mont-Saint-Guibert, Belgium; b Laboratory of Microbiology, Wageningen University, Wageningen, The Netherlands; c Clinical Microbiomics, Copenhagen, Denmark; d Metabolism and Nutrition Research group (MNUT), Louvain Drug Research Institute (LDRI), UCLouvain, Université Catholique de Louvain, Brussels, Belgium; e Walloon Excellence in Life Sciences and Biotechnology (WELBIO), WELBIO Department, WEL Research Institute, Avenue Pasteur, Wavre, Belgium; f Section of Biomolecular Medicine, Division of Systems Medicine, Department of Metabolism, Digestion and Reproduction, Imperial College London, London, United Kingdom; g Institute of Experimental and Clinical Research (IREC), UCLouvain, Université Catholique de Louvain, Brussels, Belgium; h Human Microbiome Research Program, Faculty of Medicine, University of Helsinki, Helsinki, Finland

**Keywords:** *Akkermansia muciniphila* MucT, postbiotic, prediabetes, metabolic syndrome, glycemic control, intestinal microbiota, metagenome, weight control, responder analysis

## Abstract

Pasteurized *Akkermansia muciniphila* MucT was found to improve barrier function in preclinical models and a proof-of-concept study in obese and prediabetic adults. Here, we describe the results of a double-blind placebo-controlled multicenter (Ireland and Germany) trial in 142 adults with metabolic syndrome, with or without prediabetes. The primary endpoint of whole-body insulin sensitivity (Matsuda index) did not differ after 4-months of daily administration of capsules containing 30 billion cells of pasteurized *A. muciniphila* MucT compared to placebo in the intention-to-treat subjects. Subsequent exploratory analyses showed that 3-months intake of pasteurized *A. muciniphila* MucT already improved HOMA-based hepatic insulin sensitivity in prediabetic (12%; *p* = 0.05) and 63-y-or-older-age subgroups (*p* = 0.05) while increasing post-OGTT excursion of the insulinotropic hormone glucagon-like peptide 1 (GLP-1) over placebo (*p* < 0.01). Further analysis of the gut microbiota by deep metagenomic analysis showed minor effects of the intervention but revealed that the baseline microbial composition differed from that in matched healthy adults. We found that participants with low baseline *Akkermansia* gene counts experienced significant health improvements and GLP-1 excursion after 3-months of treatment with pasteurized *A. muciniphila* MucT over the placebo. These benefits included improved insulin sensitivity (as shown by Matsuda and HOMA-S indices) and GLP-1 excursion (post-OGTT) (*p* < 0.05), reductions in body weight (*p* = 0.06) and decreased trunk fat (*p* < 0.05). In conclusion, daily supplementation with pasteurized *A. muciniphila* MucT has the potential to improve health markers in overweight or obese normo- or dysglycemic adults with the most significant improvements in subjects with low baseline intestinal *Akkermansia* levels, who are apparently truly in need of this intervention.

**Clinical trial registration no.:** NCT05114018 clinicaltrials.gov.

## Introduction

Insulin resistance is a common yet often unnoticed complication of overweight and obesity in otherwise healthy adults.^1^ Individuals with metabolic syndrome are at particularly high risk, as their bodies become less responsive to insulin. Over time, this impaired response can progress to prediabetes and eventually to type 2 diabetes mellitus. Whereas many factors contribute to this pathology, the intestinal microbiota has been found to be involved and has consequently become a target for interventions.^2^ ^,^ ^3^ ^4^ These microbial communities interact closely with their hosts through several mechanisms, including metabolizing and fermenting a wide range of compounds to generate bioactive metabolites, regulating the integrity of the gut barrier, and modulating the immune system. The ability to explore commensal gut bacteria by culture-independent and multi-omics approaches has opened new avenues to harness the potential of the human microbiome to impact host health.^4^ ^,^ ^5^ This revolution has enabled the identification of human intestinal strains with potential health-promoting properties, referred to as next-generation beneficial microbes. One of the most prominent candidates is the mucosal symbiont *Akkermansia muciniphila,* which, unlike all others, grows on host-derived mucus and has an extensive dialogue with the host.^5‐7^

Following an extensive set of preclinical experiments,^8^ ^,^ ^9^ we demonstrated the importance of *A. muciniphila* MucT in regulating host health and metabolism. We further translated these findings into a first proof-of-concept study.^10^ Supplementation with pasteurized *A. muciniphila* MucT in subjects with metabolic syndrome and insulin resistance markedly improved insulin sensitivity, plasma total cholesterol levels, markers of inflammation and gut barrier function compared with volunteers supplemented with placebo. The intervention was very well tolerated and safe. Of note, the overall gut microbiome structure remained unaffected in this human trial, implying a direct effect of pasteurized *A. muciniphila* MucT on human health. Among the potential mechanisms, we attributed part of these effects to Amuc_1100, an abundant 32 kDa protein that decorates the type IV pili of *A. muciniphila* MucT and signals to Toll-like receptor 2 (TLR2).^9^ ^,^ ^11^ The thermostable Amuc_1100 protein retained its functionality after pasteurization, and multiple preclinical models have shown that it improves barrier function, leading to a range of functional benefits.^12^ ^,^ ^13^ Another less studied protein produced by *A. muciniphila* MucT is the 84-kDa P9 protein (the product of Amuc_1631) that interacts with the intercellular adhesion molecule 2 (ICAM-2) and induces the production of glucagon-like peptide 1 (GLP-1) in mice, although its thermal stability has not been addressed.^5^ ^,^ ^12^ ^,^ ^14^

In human cohorts, a great number of health benefits have been associated with the gut levels of *Akkermansia* spp., as first discovered in prediabetic subjects using metagenomics and phylogenetic microarray approaches.^15^ This has been confirmed in multiple studies, and the link between low gut levels of *Akkermansia* and deviations including obesity, inflammatory bowel disease, liver dysfunction, and metabolic disorders has been firmly established.^5^ ^,^ ^16^ An early study showed that subjects with high intestinal baseline *A. muciniphila* were healthier than subjects with low levels that responded differentially to a dietary intervention of caloric restriction.^17^ Recently, a trial with pasteurized *A. muciniphila* MucT was found to improve weight loss maintenance and insulin sensitivity in humans with overweight and obesity—here the most prominent effects were observed in subjects with low baseline gut *Akkermansia* levels.^18^

Here, we studied the impact of the administration of pasteurized *A. muciniphila* MucT on markers of obesity and glycemia in a multicenter randomized, double-blind, placebo-controlled, parallel trial in otherwise healthy subjects with overweight or obesity, with or without dysglycemia and other features of metabolic syndrome (NCT 05114018). In addition to evaluating the primary endpoint of insulin sensitivity and various secondary health and safety outcomes, this study also analyzed the gut microbiota using deep metagenomic sequencing. This allowed us to determine how the treatment affected the microbiota composition and to assess whether the baseline microbiota profiles influenced their response to the treatment. These results indicate that the administration of pasteurized *A. muciniphila* MucT has the potential to improve parameters of glycemia in subjects with metabolic syndrome, with more pronounced health-related effects in subjects with low baseline *Akkermansia* levels on insulin sensitivity and GLP-1 response after an oral glucose tolerance test, body weight, trunk, and android fat mass.

## Methods

### Study design

We conducted a 16-week double-blind, randomized placebo-controlled parallel phase 2 study investigating the efficacy of pasteurized *A. muciniphila* MucT on insulin sensitivity, metabolic health and weight management in adults with overweight or obesity, and metabolic syndrome (ClinicalTrials.gov NCT05114018). Participants were recruited locally in Cork, Ireland, and Kiel, Germany, via volunteer databases and advertisements in local and online media from September 2020 until August 2023. Eligible participants were randomized at a 1:1 ratio to pasteurized *A. muciniphila* MucT or placebo. Randomization was stratified according to study center (Cork vs. Kiel) and the number of criteria for which eligible subjects qualify for the diagnosis of metabolic syndrome (3 or 4 criteria vs. 5).

Participants, sponsors and investigators were unaware of the trial-group assignments (treatment or placebo). The trial was approved by the local ethics committee in Cork and Kiel and was conducted according to the principles of the Declaration of Helsinki, Good Clinical Practice guidelines (the International Council for Harmonisation, ICH) E6 (R2) and all applicable local regulatory requirements. All participants provided written informed consent before any study-specific procedures were performed.

**Figure 1.**
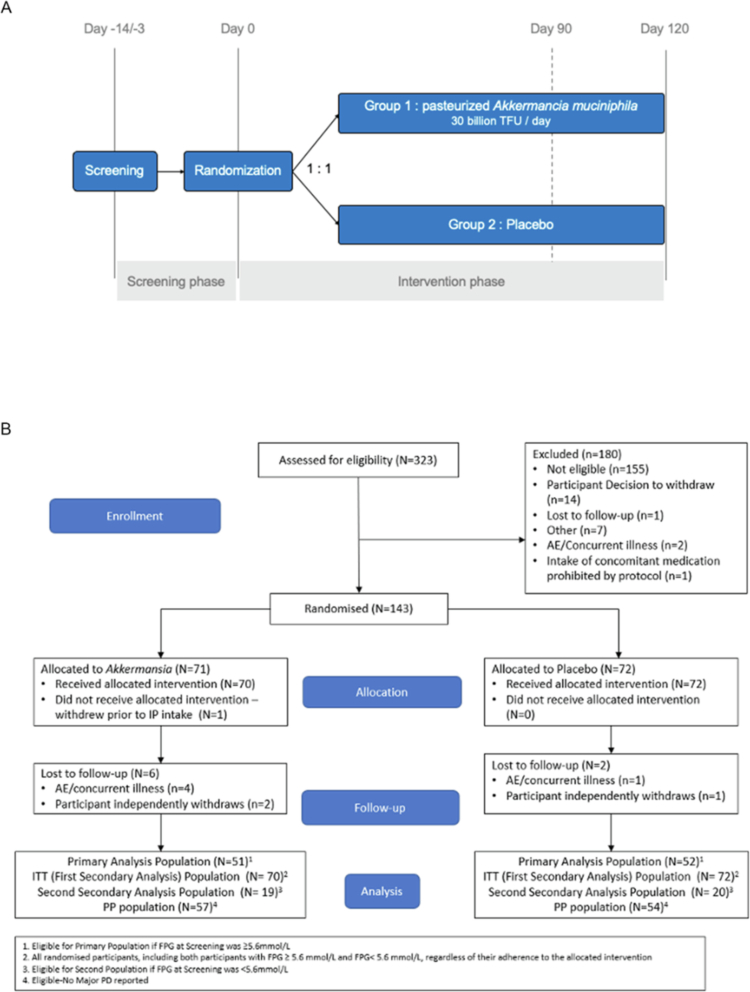
(A). Study design. All analyses were performed at D0, D90, and D120, except dual-energy X-ray absorptiometry (DEXA) and fecal microbiome analysis, which were only performed at D0 and D120. (B). Consort diagram. AE: adverse event, FPG: fasting plasma glucose, IP: investigational product, ITT: intention to treat, PP: primary population.

The study consisted of a screening period (−14 to −3 d) and a treatment period of 16 weeks (Figure 1). The participants were expected to attend 6 visits in total throughout the entire study period. It is important to note the temporary hold on recruitment during the COVID-19 pandemic in Q2 and Q3 2020 as well as Q1 of 2021.

### Study population

Eligible participants were adults (21–75 y of age) with overweight or obesity, defined as a body‒mass index (BMI) of ≥25 and <40 and who qualified for a diagnosis of metabolic syndrome according to the International Diabetes Federation (IDF 2006) criteria. All the inclusion and exclusion criteria are provided in Table S1.

A diagnosis of prediabetes or type 2 diabetes mellitus was excluded unless the participant had been unmedicated for three months prior to screening. To ensure the enrollment of subjects with dysglycemia, a protocol amendment required that the subjects had fasting hyperglycemia of ≥100 mg/dL (5.6 mmol/L).

### Intervention

The enrolled participants received daily 30 billion total fluorescent units (TFU) of pasteurized *A. muciniphila* MucT or placebo in identical capsules. The participants were instructed to consume one capsule per day, at least 15 min before breakfast, with a glass of water for the duration of the study. If the participant missed a dose in the morning, they were allowed to continue to take the missed dose later in the day (before a meal). Participants were further directed to follow their habitual diet and exercise routine.

### Investigations

#### Population characteristics

Study population demographics, including medical history (including postmenopausal status), medication use, and lifestyle characteristics, were collected via questionnaires, including a 3-d diet diary.

#### Oral glucose challenge

Fasting blood samples were taken to serve as a baseline. Then, the subjects ingested 200 mL of ready to use 75 g glucose solution within a maximum of 5 min. Venous blood draws were subsequently collected at T = −15, 0, 30, 60, 90, 120, and 180 min via an intravenous cannula for determination of plasma glucose, insulin and triglyceride concentrations.

Matsuda index and incremental area under the curve (iAUC) for both glucose and insulin as well as HOMA-S% (homeostasis model assessment of insulin sensitivity) were calculated based upon the values obtained from the 3-h oral glucose tolerance test (OGTT). HOMA-S% was calculated for the secondary endpoint using the HOMA2 calculator provided by Oxford University Innovation Ltd., Oxford, UK.

#### Biochemical analysis of blood samples and biobanking

The participants were characterized using blood plasma and serum, urine, and feces.

Serum tubes were allowed to clot for at least 20–120 min at room temperature and subsequently centrifuged at 3000 rpm at 4 °C for 10 min. They were used for clinical chemistry tests for urea, creatinine, total protein, albumin, globulins, total bilirubin, alanine aminotransferase, aspartate aminotransferase, alkaline phosphatase, gamma-glutamyl transferase, cholesterol total, triglycerides, high-density lipoprotein (HDL), direct low-density lipoprotein (LDL), calcium, phosphate, magnesium, sodium, and potassium. EDTA plasma tubes were stored on ice and rapidly centrifuged at 3000 rpm at 4 °C for 10 min. They were used for determining complete blood counts (CBC), white blood cell differentials HbA1c, and GLP-1 levels. The sodium fluoride oxalate tubes were refrigerated at 4 °C and used for glucose testing. Both serum and plasma samples were subsequently aliquoted and stored at 4 °C, −20 °C or −80 °C as defined until analysis could be performed.

Stool was collected using a sample collection kit into collection vials that were placed immediately in the home freezer and transported frozen form with cooler block to the laboratory where they were stored at −80 °C prior to further analysis.

#### GLP-1 measurements

Plasma GLP-1 concentrations were assessed in relation to the OGTT at timepoints T-15′, T + 15′, T + 30′, and T + 60′ at the baseline, D90 and D120 study visits and the area under the curve from T-15′ to T + 60′ was calculated. GLP-1 was quantified as total GLP-1, reflecting overall hormone secretion. Measurements were performed using a validated radioimmunoassay.^19^

#### Anthropometrics and body composition

Total body weight was measured using a digital medical scale at the clinical visit, and blood pressure was measured by the average of three readings (obtaining 4 and discrediting the first). Waist and hip circumference measurements were performed using a non-flexible measuring tape, with waist measurement being done in the middle of the lowest rib and the iliac crest, with the average three measurements being used. Body composition, including regional fat mass, total body mass, total body fat mass, total body fat percentage, total body lean mass or total body lean mass percentage were ascertained using dual-energy X-ray absorptiometry (DEXA). Bioelectrical impedance analysis (BIA) was also used to measure body fat mass and percentage as well as muscle mass and percentage.

#### Microbiome analysis

DNA was extracted from ~0.1 g fecal samples using the NucleoSpin 96 Stool Kit. The genomic DNA was quantified and normalized to 5 ng/μl. The DNA was fragmented, and adapter sequences were added during library construction. The concentration of the final library was measured, and the library was sequenced on a NovaSeq system (Illumina) with 2 × 150 bp read lengths. After trimming for quality and filtering for human contamination, the reads were mapped to a comprehensive gene catalog. Read mapping was performed using BWA mem (v. 0.7.17).^20^ Gene counts were derived from mapped high-quality reads assigned to annotated gene clusters. Metagenomic reads were used to determine species-level relative abundance in fecal samples at baseline and after treatment using CHAMP™.^21^ Genes were functionally annotated using EggNOG-mapper (v. 2.1.7, Diamond mode), the KEGG Orthology (KO) databases (prokaryotes), and KofamScan (eukaryotes). The KEGG module (v. 78.2) and gut metabolic module (GMM) profiles (v.1.0.7) were derived from species-level data by assigning species to modules based on ≥2/3 pathway completeness for modules with more than four components and full completeness for modules with less than four components. Module-level abundances were computed by aggregating contributions from all species associated with each module.

Microbiome diversity, species, genera, families, orders, classes, phyla, GMMs, and KEGG modules were compared between D0 vs. D120 and/or placebo vs. control using a linear modeling framework based on LinDA.^22^ Comparisons were tested both with and without adjusting for baseline abundance (when relevant), sex and age. Group-wise comparisons were tested using PERMANOVA. Paired statistical tests were done using a Wilcoxon signed rank test. For statistical testing of multiple hypotheses, the Benjamini–Hochberg (BH) method was used to control the false discovery rate (FDR) at a level of 10%.

#### Definition of subgroups with low and high *Akkermansia* spp. at baseline

*Akkermansia* gene counts at baseline in the treatment group were analyzed and used to divide the group into two subgroups of similar size with high and low *Akkermansia* spp., respectively. The median value for all the subjects was used as the cut-off value. For comparative reasons, the same division of metagenomic gene counts was applied to the baseline data of the placebo group to generate two subgroups, i.e. high and low *Akkermansia* spp. The clinical response was then studied in the different subgroups.

#### Microbiota characteristics comparison between responders and non-responders

A responder analysis was performed on three clinical markers. The responders were defined as showing a statistically significant positive change after 4 months in the Matsuda index, HOMA-s, and HbA1c, respectively (>0.73, >17, <−1.5 and *n* = 28, *n* = 36, *n* = 39, respectively). Non-responders were defined as showing a negative change in the Matsuda index, HOMA-s, and HbA1c, respectively (*n* = 59, *n* = 53, and *n* = 44, respectively). Microbiome diversity, taxonomic composition, and functional potential at baseline (D0) and D120 were compared between the responder and non-responder groups. A Wilcoxon signed-rank test was used to test for changes in abundance at D0 and D120 and for delta values (D120—D0). Changes were evaluated for alpha diversity (species richness and Shannon index), taxonomy composition at multiple levels (species, genus, family, and phylum), and functional potential (assessed through KEGG modules and GMMs). For statistical testing of multiple hypotheses, the BH method was used to control the FDR at a level of 10%.

#### Comparison of baseline microbiota characteristics between intervention group and healthy controls

Healthy controls were selected from the Clinical Microbiomics data warehouse using criteria to include only samples with read lengths >150 bp and allowing only one sample per subject. The samples were further filtered to match adults without a disease indication and the geography of the ITT samples. For Cork, samples from Ireland were used, and for Kiel, continental samples from German, Danish, Luxembourg, and French were included.

### Outcome variables

The primary endpoint was the absolute change in the Matsuda index from baseline (D0) to day 120 (D120; 4 months) by pasteurized *A. muciniphila* compared to placebo in the Intention to Treat (ITT) population, which consisted of 143 adult participants with metabolic syndrome. The key secondary endpoint was the absolute change in insulin sensitivity by homeostatic model assessment of insulin sensitivity (HOMA-S%) from baseline (D0) to D120 by pasteurized *A. muciniphila* compared to placebo. Other secondary endpoints were: HOMA-IR, HbA1c, fasting blood glucose (FBG), blood lipid levels, anthropometrics, body composition, and safety. GLP-1 blood concentration and the gut microbiome research were exploratory endpoints. Insulin sensitivity-related and other blood parameters were also measured 3 months (D90) after randomization.

### Statistical analysis

To identify differences in baseline characteristics and to verify if stratification between group allocation was successful, parameters such as sex, age and starting weight were assessed using independent *t*-tests and Mann‒Whitney U tests for continuous data.

We estimated that a sample of 53 participants in each group would provide the trial with 80% power to detect a minimal clinically important difference in the primary end point, i.e. change in the Matsuda index of 1.35 at a one-sided alpha level of 0.05. For secondary endpoints, there is no pre-defined multiple testing procedure. *p*-values resulting from these analyses only have an exploratory intent.

The primary analysis of the Matsuda index was performed using a mixed model for repeated measurements (MMRM), with the absolute change from baseline in the Matsuda index on the log scale as the response (dependent) variable and the baseline Matsuda index, study center (site), metabolic syndrome criteria (<5 vs 5, factor as used at randomization), treatment arm, timepoint, and treatment-by-timepoint interaction as covariates. Also, the secondary endpoints and exploratory GLP-1 were analyzed using MMRM with the parameter at baseline, the study center, and the metabolic syndrome criteria as covariates. The GLP-1 values were log-transformed for analysis. No formal interim analysis was performed.

The subgroup analyses were performed on the following subgroups for the change from baseline in the Masuda index, HOMA-S, and HbA1c as well as the other secondary endpoints: site, gender, age (<63 vs ≥ 63y), post-menopausal status by reporting, and abundance level of *Akkermansia* spp. in stool at baseline (<median gene count vs> median gene count).

## Results

### Trial participants, baseline characteristics, and compliance

**Figure 2.**
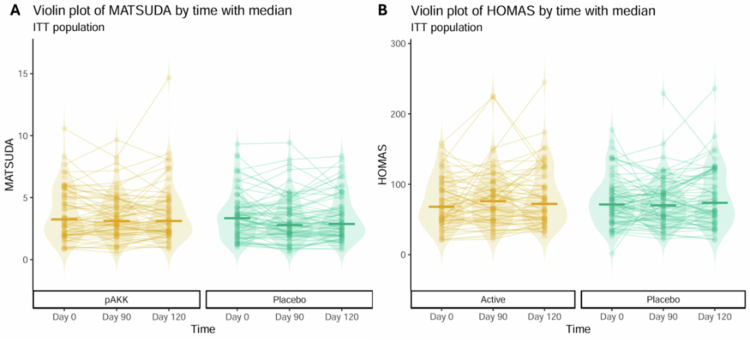
(A). Matsuda index and (B). HOMA-S at baseline, Day 90 and Day 120 in the ITT population. HOMA-S: homeostatic model assessment for insulin sensitivity.

The participants in Cork (Ireland) and Kiel (Germany) were equally randomized to one of the two study arms, one receiving a daily capsule containing 30 billion TFU of pasteurized *A. muciniphila* MucT and maltodextrin (treatment), and the other receiving a daily capsule with maltodextrin as a Placebo (Figure 2). This ITT population consisted of 143 adult participants (aged 24–75 y, average 61 y) with metabolic syndrome (insulin resistance, obesity), who were balanced in terms of sex (55.9% females), site (54% in Cork, 46% in Kiel), anthropometric and metabolic markers (insulin sensitivity, BMI, body composition) as well as postmenopausal status (39/33% in the active/placebo arm respectively) (Table 1). These baseline characteristics did not differ across groups. Among the 143 randomized participants, one randomized participant was found not to be eligible before the administration of any investigational product (IP) and was therefore removed from the statistical analysis (Figure 1). The safety population included all participants who had taken at least one dose of the IP. All 142 participants (*n* = 70, *Akkermansia* and *n* = 72, Placebo) were exposed to at least one dose of the IP.

**Table 1.** Baseline characteristics.

Characteristics ITT population	*A. muciniphila* *n* = 70	Placebo *n* = 72
Site: Cork/Kiel	39 (56%)/31 (44%)	38 (53%)/34 (47%)
Age (y)	62 ± 11	60 ± 10
Women (no (%))	37 (53%)	43 (60%)
Postmenopausal women (no (%))	27 (39%)	24 (33%)
Body weight (kg)	94.61 ± 14.07	91.96 ± 14.64
BMI (kg/m^2^)	32.34 ± 3.79	31.54 ± 3.51
Body fat percentage (%)	41.12 ± 7.57	40.20 ± 6.69
Total body fat mass (kg)	37.79 ± 9.10	35.55 ± 7.82
Total body lean mass (kg)	54.12 ± 10.45	53.14 ± 11.12
Waist circumference (cm)	106.25 ± 11.00	103.61 ± 11.69
Hip circumference (cm)	112.00 ± 8.97	110.15 ± 7.71
Matsuda index	3.74 ± 2.04	3.52 ± 1.90
HOMA-S%	74.11 ± 33.86	75.98 ± 35.29
HOMA-IR	3.32 ± 2.00	3.26 ± 1.68
Fasting blood glucose ≥ 5.6 mmol/L (no (%))	51 (73%)	52 (72%)
HbA1c (mmol/mol)	36.59 ± 3.95	35.85 ± 3.78
Total cholesterol (mmol/L)	5.07 ± 1.18	5.30 ± 1.21
HDL-Cholesterol (mmol/L)	1.32 ± 0.30	1.34 ± 0.26
LDL-Cholesterol (mmol/L)	3.27 ± 1.16	3.53 ± 1.17
Triglycerides (mmol/L)	1.46 ± 0.73	1.59 ± 0.83

Data expressed as the observed means (±standard deviation or percentage of subjects). No statistical difference was found between the two groups. BMI: body mass index, HbA1c: hemoglobin A1C, HDL: high-density lipoprotein, HOMA-IR: homeostatic model assessment for insulin resistance, HOMA-S: homeostatic model assessment for insulin sensitivity, ITT: intention to treat, LDL: low-density lipoprotein, no: number.

The initial target was to include subjects with insulin resistance, but due to recruitment challenges during the COVID epidemic, this criterion was adapted, and the ITT group also included obese subjects with normal FBG levels (Table 1). Hence, the primary analysis population consisted of 103 subjects with metabolic syndrome with prediabetes and included 51 and 52 subjects in the active and control arm, respectively, while the ITT analysis population also included the non-prediabetics (Figure 1).

A total of 87.5% and 87.3% of all participants in the active and placebo arm, respectively, were fully compliant with the protocol. The compliance with investigational product consumption in the study was excellent (over 99% in both arms based on the returned supply of the investigational product).

The diet history did not reveal significant changes in caloric intake between both arms.

### The impact of pasteurized *A. muciniphila* MucT supplementation on insulin sensitivity and other metabolic health parameters

The overall results of the study showed a small, non-significant improvement in whole-body insulin sensitivity, as determined by the Matsuda index of up to 5 and 7% after 3 months of intervention with pasteurized *A. muciniphila* MucT versus placebo in the prediabetic (primary analysis) and overall (ITT) metabolic syndrome population, respectively. However, this effect diminished after 4 months, concluding to a negative primary study outcome (Figure 2).

Hence, we report here on the exploratory analysis results of the secondary and exploratory endpoints performed as *post hoc* analyses, including subgroup analyses such as the number of participants and their individual characteristics, which allowed for subgroup analysis and segmentation based on the baseline microbiota. The main subgroups studied were defined by the presence of prediabetes, postmenopausal status, age, and baseline *Akkermansia* gut levels (see below).

**Figure 3.**
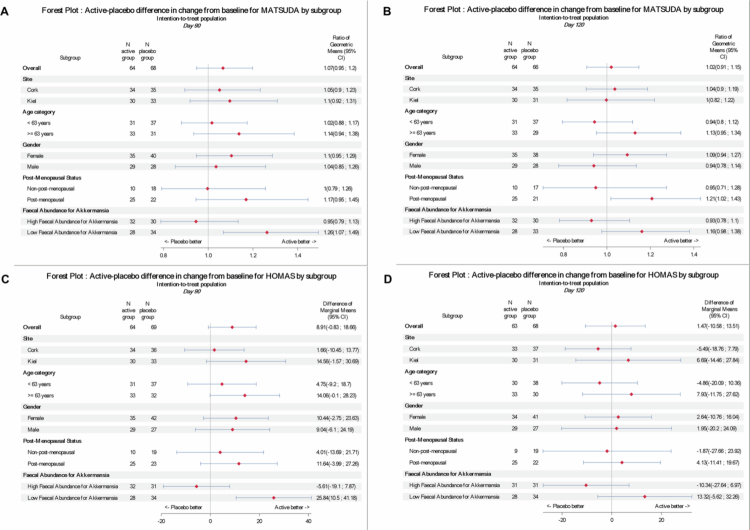
Forest plots of (A, B). Whole-body insulin sensitivity (MATSUDA index) and of (C, D). HOMA-S% sensitivity (Oxford calculator) showing difference between baseline and D90 or D120, respectively. D = day; HOMA-S: homeostatic model assessment for insulin sensitivity.

Analysis of the baseline characteristics revealed a significant number of postmenopausal women (Table 1). Perimenopause is associated with changes in weight, body composition and energy balance and an increased risk for insulin resistance.^23^ Hence, we first focused on post-menopausal women in the subgroup analyses. Insulin sensitivity, determined as Matsuda index, increased significantly after 4 months of intervention with pasteurized *A. muciniphila* MucT over placebo in postmenopausal women (*n* = 46) (+21%; *p* = 0.031, in the ITT population). Moreover, when all participants, both male and female, were 63 y or older were considered (*n* = 62), their insulin sensitivity, determined by the Matsuda index, tended to increase by up to 14% and 13% when taking pasteurized *A. muciniphila* MucT compared to placebo on D90 and D120, respectively (*p* = 0.192 and *p* = 0.159, respectively) (Figure 3, Tables S2 and S3).

Interestingly, supplementation with pasteurized *A. muciniphila* MucT led to an almost significant improvement in HOMA-based insulin sensitivity. In the prediabetic subgroup, insulin sensitivity increased by 12% after 3 months compared with placebo (*p* = 0.054; 48 vs. 51 participants). A similar trend was observed in the overall ITT population, with a 9% increase in HOMA-S% after 3 months (*p* = 0.07) (Figure 3, Table S2).

Regarding glycemic control, HbA1c levels in the pasteurized *A. muciniphila* MucT group showed a small but consistent reduction over 4 months of intervention compared with placebo (0.28–0.44 mmol/mol or 0.1% in NGSP units difference^24^ in both the primary and ITT populations). At the lipid level, total cholesterol, LDL, and triglyceride serum values did not change significantly between the two study groups after 3 or 4 months in the prediabetic or ITT population. Unexpectedly, HDL-cholesterol serum levels decreased in the pasteurized *A. muciniphila* MucT treated group versus placebo after 3 months but did not differ anymore after 4 months in both groups (Table 2). In addition, the liver function tests did not differ between the two study groups in the primary or ITT population. Kidney function measured by the estimated glomerular filtration rate (eGFR) and plasma creatinine levels, tended to improve after 4 months treatment with pasteurized *A. muciniphila* MucT compared to placebo (eGFR *p* = 0.12, creatinine *p* = 0.06). Interestingly, in postmenopausal women, this difference Compared to placebo was significant (eGFR *p* = 0.031, creatinine *p* = 0.008) (Figure S1B).

**Table 2.** Estimated differences by pasteurized *A. muciniphila* vs. placebo in metabolic health‐related endpoints.

Endpoint	D0–D90	*p*-value	D0–D120	*p*-value
HOMA-IR ITT	−0.244 (−0.731 to 0.243)	0.323	−0.150 (−0.619 to 0.318)	0.425
Fasting plasma glucose (mmol/L) ITT	0.001 (−0.143 to 0.144)	0.991	0.084 (−0.109 to 0.276)	0.391
HbA1c (mmol/mol) ITT	−0.304 (−1.19 to 0.582)	0.499	−0.28 (−1.383 to 0.823)	0.612
Postprandial glycemia (3-h OGTT) ITT	0.182 (−0.182 to 0.547)	0.324	0.244 (−0.154 to 0.642)	0.228
Postprandial insulinemia (3-h OGTT) ITT	−2.906 (−10.546 to 4.735)	0.453	−2.914 (−10.849 to 5.022)	0.469
Postprandial triglyceridemia (3-h OGTT) ITT	0.161 (−0.138 to 0.460)	0.289	0.140 (−0.056 to 0.336)	0.160
Total cholesterolemia (mmol/L) ITT	−0.023 (−0.320 to 0.273)	0.876	0.072 (−0.150 to 0.294)	0.523
HDL-cholesterolemia (mmol/L) ITT	−0.060 (−0.119 to −0.002)	**0.043**	0.028 (−0.027 to 0.083)	0.318
LDL-cholesterolemia (mmol/L) ITT	0.04 (−0.191 to 0.271)	0.735	0.041 (−0.161 to 0.243)	0.689
Triglyceridemia (mmol/L) ITT	0.157 (−0.175 to 0.488)	0.352	0.133 (−0.090 to 0.357)	0.239

Data are expressed as estimated means (95% confidence intervals). Mean changes were estimated from a mixed model for repeated measures with time, product and time‒product interactions as variables adjusting for the baseline parameter measured, metabolic syndrome criteria and site HbA1c: hemoglobin A1C, HDL: high-density lipoprotein, HOMA-IR: homeostatic model assessment for insulin resistance, ITT: intention to treat, LDL: low-density lipoprotein. Significant *p*-values are indicated in bold.

**Figure 4.**
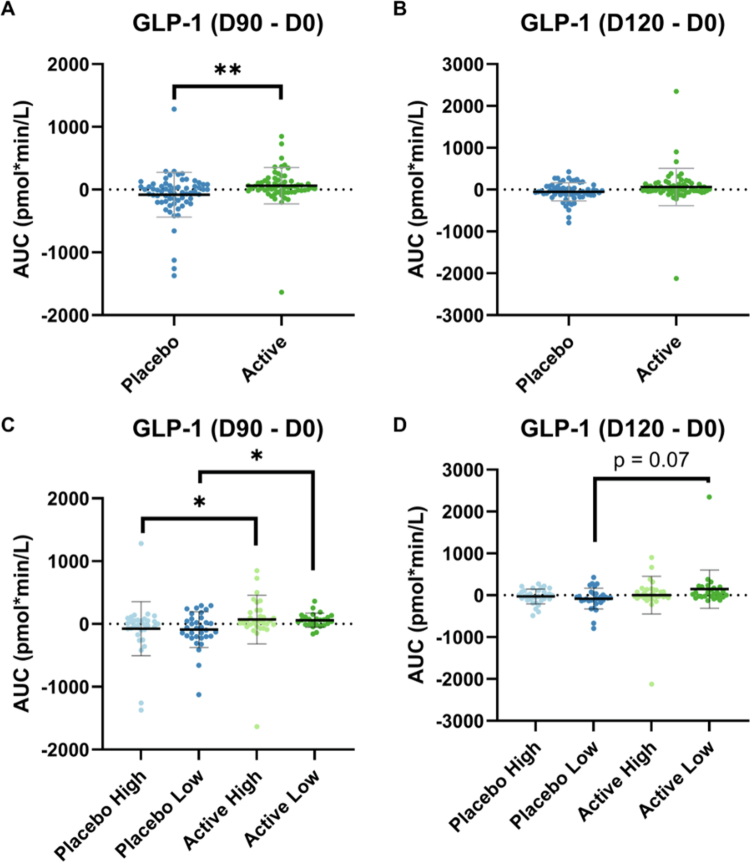
(A, B). Difference in GLP-1 response (AUC) to OGTT after 3 (D90) or 4 (D120) months of treatment. (C, D). Difference in the GLP-1 response to the OGTT after 3 (D90) or 4 (D120) months of treatment in subjects with high or low fecal levels of *Akkermansia* spp. *p < 0.05, **p < 0.01. AUC = area under the curve; D = day.

The plasma GLP-1 levels in response to the OGTT at D0 (baseline), Days 90 and 120 of the intervention showed a significant increase in the area under the curve (AUC) at D90 in the presence of pasteurized *A. muciniphila* MucT compared to placebo in the ITT population (Figure 4, Table S3). This effect was less pronounced after 4 months of intervention. Looking into the subgroups by baseline *Akkermansia* levels, the AUC of GLP-1 at D90 was similarly increased in the low and high baseline groups in the active arm vs. placebo. However, at D120, a non-significant increase tended to persist only in the subgroup with low baseline *Akkermansia* levels (*p* = 0.07, Figure 4, Table S3).

With respect to the anthropometric and body-composition outcomes, body weight and waist circumference showed no differences between the two study groups in either the primary or ITT populations. Hip circumference was the only anthropometric measure that changed significantly, showing a small but statistically significant increase of almost +1 cm in the pasteurized *A. muciniphila* MucT group (*p* < 0.05) at D90 in the ITT population and at D120 in the prediabetic population (Table 3).

**Table 3.** Estimated differences by pasteurized *A. muciniphila* vs. placebo in anthropometric and body composition endpoints in ITT population and in ITT population with low fecal *Akkermansia* spp. level at baseline.

Endpoint	ITT				Low-*Akkermansia*		
	D0–D90	*p*-value	D0–D120	*p*-value	D0–D120		*p*-value
Body weight (kg)	0.024 (−0.711 to 0.76)	0.948	−0.070 (−0.971 to 0.831)	0.878	−1.298 (−2.640 to 0.045)		0.058
Total body fat mass (kg)	–	–	−0.326 (−1.235 to 0.584)	0.48	−1.0644 (−2.128 to −0.001)		0.05
Trunk fat mass (kg)	–	–	−0.347 (−0.969 to 0.273)	0.27	−0.796 (−1.553 to −0.04)		0.039
Android fat mass (kg)	–	–	−0.111 (−0.242 to 0.02)	0.095	−0.254 (−0.440 to −0.067)		0.009
Gynoid fat mass (kg)	–	–	−0.041 (−0.233 to 0.151)	0.673	−0.219 (−0.467 to 0.029)		0.082
Total body lean mass (kg)	–	–	−0.109 (−0.738 to 0.52)	0.733	−0.265 (−0.954 to 0.424)		0.444
Waist circumference (cm)	0.081 (−0.829 to 0.991)	0.860	−0.105 (−1.127 tot 0.918)	0.84	−0.927 (−2.542 to 0.688)		0.255
Hip circumference (cm)	0.801 (0.007 to 1.595)	0.048	0.997 (0.163 to 1.830)	**0.02**	Low-*Akkermansia*	0.256 (−1.024 to 1.536)	0.984
					High-*Akkermansia*	1.432 (0.301 to 2.562)	**0.014**

Data are expressed as estimated means (95% confidence intervals). Mean changes were estimated from a mixed model for repeated measures with time, product and time‒product interactions as variables adjusting for the baseline parameter measured, metabolic syndrome criteria and site. Significant p-values are indicated in bold.

### Metagenome-based microbiota composition at baseline and after intervention with pasteurized *A. muciniphila* MucT

To study the impact of the pasteurized *A. muciniphila* MucT intervention on the gut microbiota, we determined species-level relative abundances in fecal samples based on metagenomic reads at baseline and after treatment (D120). No significant shift in species richness or the Shannon alpha diversity index was found after 4 months treatment when considering all the subjects (Supplementary Figure S2). Species richness showed a small but significant decrease over time in the treatment group (−14.3 ± 6.9, *p* < 0.05), while no other microbiome feature was significantly different in either of the two intervention groups. In addition, no difference in beta diversity was found after 4 months of treatment, and there was no difference in Bray‒Curtis dissimilarity between D0 and D120 for the placebo versus the treatment group, indicating similar variability over time in the two intervention groups.

There were also no changes over time in individual species, genera, families, orders, classes, phyla, GMMs, and KEGG modules except for the genus *Megasphaera,* which showed a small but significant increase over time in the treatment group but not in placebo. When comparing the placebo and treatment, we found that only the species *Pauljensenia* sp018375675 and *Intestinimonas stercoravium* were differentially abundant between the placebo and treatment at D120, with *I. stercoravium* being higher in the placebo and *P.* sp018375675 being lower in the placebo compared to the treatment group (Figure S2, Supplementary materials).

### Microbiota characteristics comparison between responders and non-responders

We stratified the subjects by clinical response (Matsuda index, HOMA-s, and HbA1c, respectively) and defined responders (statistically significant improvement) and non-responders (any negative change). Microbiome diversity, taxonomic composition, and functional potential at baseline (D0) and D120 were compared between responders and non-responders. Very limited differences were found. Together, the tests for the abundance of species, genera, phyla, GMMs, and KEGG modules that were significantly different between responders and non-responders at baseline and D120, as well as for delta abundance, altogether detected 5 species, 6 genera, 1 phylum, 2 GMMs, and 1 KEGG module with statistically significant changes (Figure S2, Supplementary materials). When analyzing delta abundance values between the responder and non-responder groups, the KEGG module M00708, associated with multidrug resistance and the PatAB transporter, showed a significant increase in the non-responder group. *Akkermansia muciniphila* did not exhibit significant differences in any of the comparisons between responder and non-responder groups, but we noticed a trend towards lower baseline *Akkermansia* levels in responders (Supplementary materials). Based on this, we decided to (1) compare the microbiome of the studied population to that of healthy volunteers and (2) to investigate the influence of baseline levels of *Akkermansia* on response to treatment.

### Baseline microbiota characteristics of the intervention group

The in-depth metagenomic analysis of fecal samples from the ITT participants enabled us to examine whether their gut microbiomes displayed characteristic features known to differ between prediabetic and healthy individuals, particularly the commonly reported reduction in the relative abundance of *Akkermansia* species.^25^ To correct for potential regional differences, the gut microbiota composition of healthy and ITT subjects was compared by selecting region-matched adult healthy controls from Cork (63 healthy with 65 ITT subjects) or Kiel (62 healthy with 64 ITT subjects) and analyzing these with the same bioinformatics pipeline as the ITT samples.

A Principal coordinate analysis clustering by using weighted UniFrac distances showed clear and significant separation of the ITT subjects and their geographically matched healthy controls at both sites (Figure S3). The gut microbiota diversity of the healthy subjects in Kiel was higher than that of the ITT subjects while no differences in diversity were observed between the Cork ITT and healthy controls selected (Figure S4). Apparently, the Cork groups differed more evenly than the Kiel groups and a total of 7 taxa were found to diverge significantly between the healthy and ITT subjects, whereas this number amounted to only 4 in Kiel (Figure S4). No significant differences were observed in the relative abundance of *Akkermansia* spp. Although, its relative abundance tended to be higher in the ITT compared to the healthy control at both sites (*p* = 0.1). Remarkably, the relative abundance of *Gemmiger* spp. (notably *Gemmiger formicilis* in Cork), a known butyrate and formate producer, and *Faecalibacterium* spp. (notably *Faecalibacterium longum* in Kiel, another well-known and abundant butyrate producer, was found to be significantly increased in the ITT subjects at both sites, whereas only *Phocaeicola* and *Lachnospira* spp. were increased in the healthy controls in Cork and Kiel, respectively.

### Supplementation with pasteurized *A. muciniphila* MucT improves insulin sensitivity in volunteers with low baseline *Akkermansia* spp.

**Figure 5.**
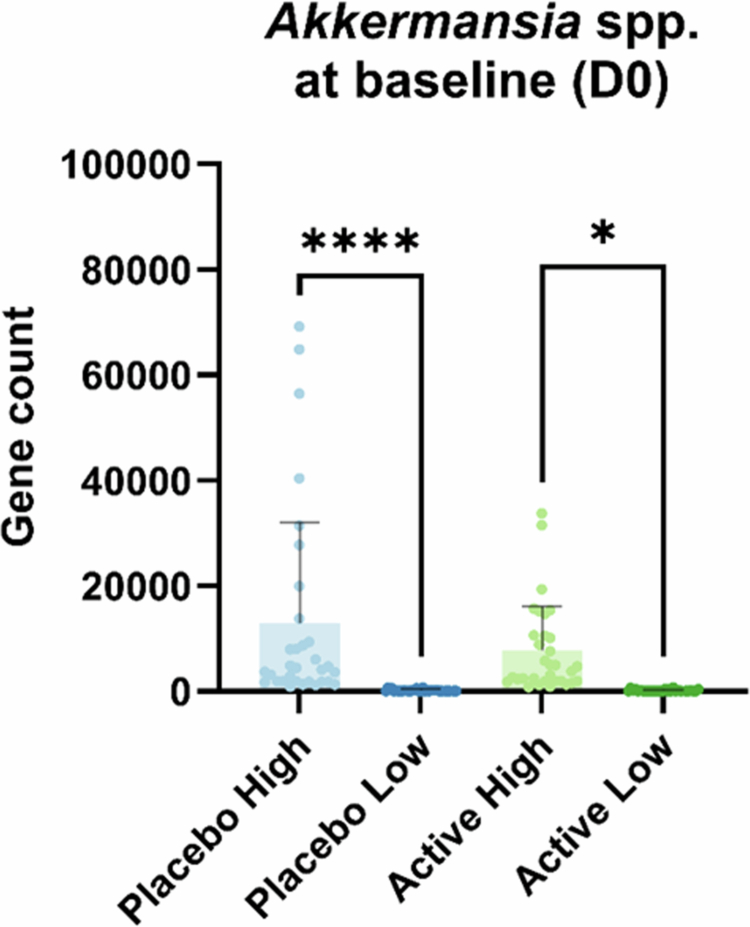
Baseline *Akkermansia* spp. levels as defined by genus level gene counts in high and low baseline placebo and active subgroups. One-way ANOVA with multiple comparisons was used to compare Placebo high vs. low, Active high vs. low, Placebo high vs. Active high and Placebo low vs. Active low. *p < 0.05, ****p < 0.0001. D = day.

Fecal baseline levels of *Akkermansia* spp. in the ITT subjects were unexpectedly not lower than those of healthy controls. It is known that these levels may determine the outcome of dietary interventions.^17^ In addition, overweight or obese subjects with a low absolute abundance of fecal *A. muciniphila* showed the best body weight maintenance and cardiometabolic benefits from treatment with pasteurized *A. muciniphila* MucT after a period of weight loss.^18^ Therefore, we divided the treatment group in two subgroups of similar size, with low and high levels of *Akkermansia* spp. at baseline, respectively. We used metagenome-derived *Akkermansia* spp. gene counts as an estimate of absolute fecal levels of *Akkermansia* spp. and used the median value (median gene count: 772) to divide the subjects into high and low baseline subgroups (Figure 5). For comparative reasons, the same division was applied to the placebo group. The average relative abundances of *Akkermansia spp.* in each subgroup were 0.05% ± 0.06% and 0.04% ± 0.08% (low placebo and low treatment, respectively) and 2.81% ± 3.74% and 2.73% ± 3.35% (high placebo and high treatment, respectively). This division into subgroups allowed us to assess in an exploratory nature the impact by pasteurized *A. muciniphila* MucT on metabolic parameters, anthropometrics and body composition in circumstances of low abundance of *Akkermansia* spp. at baseline. The baseline characteristics did not differ between the subgroups (Table S4).

**Figure 6.**
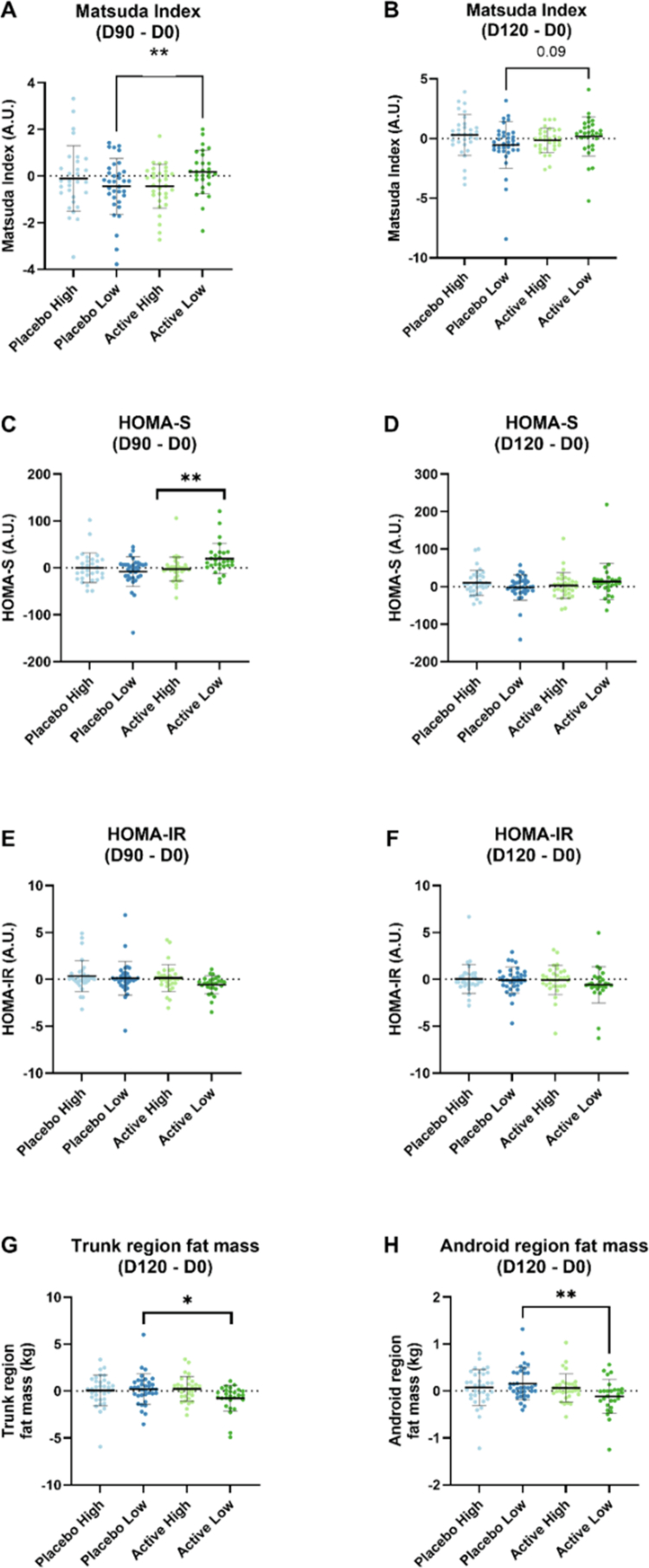
(A, B). MATSUDA, (C, D). HOMA-S, (E, F). HOMA-IR changes after 3 (D90) or 4 (D120) months of treatment in subjects with high or low fecal levels of *Akkermansia* spp. (G). Trunk region fat mass and (H). Android region fat mass changes after or 4 (D120) months of treatment in subjects with high or low fecal levels of *Akkermansia* spp. A.U.: Arbitrary Unit, HOMA-IR: homeostatic model assessment for insulin resistance, HOMA-S: homeostatic model assessment for insulin sensitivity. *p < 0.05, **p < 0.01.

In participants with low baseline fecal *Akkermansia* spp. abundance, supplementation with pasteurized *A. muciniphila* MucT led to an important improvement in insulin sensitivity. Specifically, the Matsuda index increased by 26.3% after 3 months (*p* = 0.007) and by 16.1% after 4 months (*p* = 0.09) compared with placebo (Figure 6). HOMA-S and HOMA-IR followed a similar pattern, showing significant improvements after 3 months, although these effects the improvements were no longer statistically significant at 4 months (Figure 6). The insulin-sensitizing effect in this low-*Akkermansia* group was further supported by the plasma glucose and insulin iAUC, which indicated lower insulin levels for the same glucose levels in the treatment group compared with placebo (Table S5). The *Akkermansia* abundance level did not affect the relatively neutral impact of pasteurized *A. muciniphila* MucT on HDL- and LDL-cholesterol and triglyceride levels.

In participants with low baseline *A. muciniphila* levels, the total body weight decreased more in the group receiving pasteurized *A. muciniphila* MucT than in the placebo group at both timepoints. A similar pattern was observed for waist circumference. Conversely, among participants with high baseline *A. muciniphila* levels, the hip circumference showed a small but significant increase in the pasteurized *A. muciniphila* MucT group compared with the placebo (Table 3). When considering the body composition as determined by dual-energy X-ray absorptiometry at 4 months, the same moderating effect of the pasteurized *A. muciniphila* MucT compared to placebo was observed for total body mass, total body fat mass and, to a lesser extent, lean mass in the low *Akkermansia* abundance group (Table 3). At the 4-month timepoint, the tendency for improvement of the insulin sensitivity was accompanied by a significant decrease in trunk and android region fat mass in the subjects with low baseline *Akkermansia* spp. receiving pasteurized *A. muciniphila* MucT compared to placebo (Figure 6).

### Tolerability and safety data

The intake of the investigational product (daily 30 B TFU of pasteurized *A. muciniphila* MucT) was confirmed to be safe and very well tolerated during the study. The overall incidence of adverse events (AEs) was similar between the subjects who received pasteurized *A. muciniphila* MucT and placebo. A total of 104 out of 142 participants (73%) experienced at least one AE during the study, 53 in the placebo and 51 in the active arm (Table S6). Most AEs were mild or moderate, with just 4 of severe intensity. One participant developed a serious AE (pneumonia) in the placebo group, none in the pasteurized *A. muciniphila* MucT group. Two participants in the latter group discontinued the investigational product, one in the context of moderate leg edema and one with mild abdominal discomfort.

Noting that the study was running during the COVID-19 pandemic, the two most frequent AEs were Corona infection and nasopharyngitis, the incidence of which almost doubled in the placebo group compared to the pasteurized *A. muciniphila* MucT group. The relative risk for any infection in subjects that received pasteurized *A. muciniphila* MucT was 29% lower than that in those who received placebo (*p* = 0.120).

Apart from infections, the most commonly occurring AEs in both study arms belonged to the System Organ Class (SOC) of Gastrointestinal Disorders. The AEs in this SOC were mild to moderate in nature and occurred in 25 of the participants in the pasteurized *A. muciniphila* MucT group (36%), versus 17 in the placebo group (24%) and consisted merely of dyspepsia, abdominal discomfort, flatulence and altered bowel movements. 9 of the 12 subjects who presented with an episode of hypertension or increased blood pressure as AE had pre-existing hypertension in their medical history, and all recovered. There was no statistical difference between the two arms for any of the most frequent AEs.

In the context of the safety laboratory and vital parameters, there were no remarkable differences between the two arms. All the mean values were within the normal range apart from the blood pressure values. At the cardiovascular level, we observed that the average blood pressure at baseline across the arms was in the high normal range, as is often observed in subjects with metabolic syndrome.^26^

## Discussion

This randomized, double-blind, placebo-controlled study evaluated whether supplementing pasteurized *A. muciniphila* MucT influences glucose and lipid metabolism, body weight, and body composition. Although whole-body insulin sensitivity did not differ between the treatment and placebo groups, participants who received pasteurized *A. muciniphila* MucT showed a trend toward improved liver- and pancreas-derived insulin sensitivity (HOMA-S%, calculated with HOMA2). This nearly significant improvement was observed both in the overall study population and specifically among participants with prediabetes after three months of supplementation with pasteurized *A. muciniphila* MucT.

Importantly, among the postmenopausal women who represented 39% of the total study population, the improved insulin sensitivity (as determined by the Matsuda index as well as HOMA-IR) reached significance after 4 months of intervention with pasteurized *A. muciniphila* MucT compared to placebo. This illustrates the great potential in improving metabolic conditions in menopausal women, who have an increased risk for insulin resistance and cardiometabolic health.^27^ It is well known that ageing and menopause are associated with an altered gut microbiome.^27‐29^ This includes the depletion of *Akkermansia* spp. and this is in line with the major conclusion of our study that subjects with a low gut level of *Akkermansia* will benefit most from the intake of pasteurized *A. muciniphila* MucT as will be discussed below. Moreover, we also detected a significantly improved renal function under the influence of pasteurized *A. muciniphila* MucT in postmenopausal women. Kidney function has been associated with gut barrier dysfunction, and there are reports in rodent models that suggest that a beneficial effect is exerted by *A. muciniphila* species on renal function.^30^

Enteroendocrine (EE) cells are present throughout the gastrointestinal tract in the gut mucosa, and L-type EE cells synthesize and secrete key hormones such as peptide YY (PYY), glucagon-like peptide-1 (GLP-1), and glucagon-like peptide-2 (GLP-2). These hormones play crucial roles in regulating gut motility, satiety, and glucose metabolism. GLP-1, an incretin hormone, facilitates glucose homeostasis by stimulating insulin secretion, inhibiting glucagon secretion, and slowing gastric emptying, thereby promoting satiety and weight loss.^31^ Administration of pasteurized *A. muciniphila* MucT cells increased the post-OGTT excursion of the insulinotropic hormone GLP-1 over placebo in parallel with the improvement in insulin sensitivity by HOMA-S% after three months of intervention. This observation was further emphasized in the low *Akkermansia* subgroup and suggested that pasteurized *A. muciniphila* MucT may contribute to improving glucose metabolism and lowering cardiometabolic risk through modulation of the incretin GLP-1 as well as through direct interaction between the postbiotic and the efferent nervous system (gut‒brain axis). Of note, in our earlier proof-of-concept study in overweight and obese volunteers, an increased level of serum GLP-1 over placebo was also observed after treatment with pasteurized *A. muciniphila* MucT but this was not significant, possibly due to the low number of subjects.^10^ The role of the P9 protein (the product of Amuc_1631) that induces GLP-1 production in mice has to be further studied, as it is not known whether it is secreted, is gut-stable and retains its function after pasteurization.^12^ ^,^ ^14^

It has been well described that the gut microbiota composition is involved in determining the metabolic response of obese or otherwise compromised subjects to dietary interventions.^32^ ^,^ ^33^ Similarly, improvement of insulin sensitivity in metabolic syndrome subjects by a microbial therapy was found to be driven by baseline gut microbiota composition (Kootte et al 2017). Notably, the contribution of *Akkermansia* spp. has been found to be relevant, and subjects with high intestinal baseline *A. muciniphila* were found to be healthier than subjects with low levels that responded differently to a dietary intervention.^17^ Hence, we characterized the gut microbiota composition of the subjects in our cohort by deep metagenomic analysis, which allowed identification at the species level. The 4-month intervention with pasteurized *A. muciniphila* MucT showed only very limited effects on the gut microbiota composition. No effect on the relative abundance of *A. muciniphila* was noted, while only the genus *Megasphaera*, a known propionate producer, showed a small but significant increase over time in the treatment group. In addition, abundance of *Pauljensenia* sp018375675 and *I. stercoravium* were significantly different between the placebo and treatment at D120, with *I. stercoravium* being higher and *P.* sp018375675 being lower in the placebo compared to treatment, respectively. Their relative abundances were very low and hence a metabolic effect is unlikely (Supplementary Figure S2). This is in line with an earlier study where no effect of pasteurized *A. muciniphila* MucT intake was observed in the total community by using 16S rRNA amplicon sequence analysis.^10^ The absence of a community effect can be rationalized as the administered bacteria are pasteurized and not capable of replicating, contributing to an only marginal effect upon reaching the colon. The proposed site of action is in the upper intestinal tract, as has been shown in preclinical experiments with pasteurized *A. muciniphila* MucT where the cell-envelope bound and pasteurization-resistant Amuc_1100 protein, with well-established TLR2 agonist activity, has been found to be capable of improving barrier function by increasing tight junction gene expression in the jejunum and ileum.^9^ Earlier trials have shown that decreased gut permeability effected by the intake of pasteurized *A. muciniphila* MucT resulted in decreased levels of circulating lipopolysaccharide, a well-known inflammatory endotoxin, in both mice and humans.^9^ ^,^ ^10^

Remarkably, the subjects in our cohort, both in Kiel and Cork, showed a gut microbiota composition that differed from that of matched healthy subjects from similar regions, as analyzed with the same pipeline (Figure S3). While unavoidable technical differences such as sampling and extraction may explain some variations, we noted that microbial signatures often associated with healthy subjects, such as high levels of butyrate-producing *Faecalibacterium* spp., were unexpectedly increased in the subjects in our cohort (Figure S4). Similarly, a tendency toward increased relative levels of *A. muciniphila* were observed, contrary to the expectation that metabolically compromised subjects have reduced levels of *Akkermansia* spp.^25^ Hence, we decided to elucidate the net effect of the intake of pasteurized *A. muciniphila* MucT and performed a sub-analysis in the subjects with low or high abundance of *Akkermansia* spp. at the start of the clinical trial. This assumption was grounded in the idea that individuals with low gut levels of *Akkermansia* spp. would benefit the most from the intervention. Notably, consuming pasteurized *A. muciniphila* MucT led to a significant improvement in both whole-body and liver insulin sensitivity, as measured by the Matsuda index and HOMA-IR, compared with placebo in participants who initially had low gut levels of *Akkermansia* species. These metabolic effects were accompanied by beneficial anthropometric and cardiovascular effects by pasteurized *A. muciniphila* MucT compared to placebo, such as a lower total body weight, waist circumference, total body fat mass, android and trunk region fat mass, which may suggest the involvement of the visceral fat mass in the underlying mechanism of action, as well as an importantly lower diastolic blood pressure. Interestingly, participants who initially had low levels of fecal *Akkermansia* spp. showed a significant reduction in android and trunk fat mass, which occurred alongside a marked improvement in insulin sensitivity. This is in line with the effects observed in preclinical studies where pasteurized *A. muciniphila* MucT was found to increase whole-body energy expenditure and fecal energy excretion in diet-induced obesity.^34^

Various studies have reported the levels of *Akkermansia* spp. in different geographic regions around the world, and a recent meta-analysis reported that, depending on the country, an estimated 38% to 86% of individuals have no detectable *A. muciniphila.* ^35^ Our findings therefore support the idea that people lacking detectable levels of this bacterium may be particularly likely to benefit from treatment with pasteurized *A. muciniphila* MucT.

Our observations fit very well with our earlier data obtained with pasteurized *A. muciniphila* MucT in diet-induced obesity-mice: these preclinical data reproducibly demonstrated that both live and pasteurized *A. muciniphila* MucT can enhance gut barrier function, modulate inflammatory tone, and improve metabolic control, including body weight and insulin sensitivity.^9^ ^,^ ^34^ Moreover, a recent study in a small number of Chinese subjects with overweight/obesity or type 2 diabetes that were subject to an intervention with the live *A. muciniphila* strain AKK-WST01 also showed metabolic benefits that were partly dependent on baseline intestinal levels of *Akkermansia* spp.^36^ Very recently, we showed that the administration of pasteurized *A. muciniphila* MucT improved weight loss maintenance and insulin sensitivity in humans with overweight and obesity with the most prominent health benefits in subjects with low baseline gut *Akkermansia* levels.^18^ This all explains why we noted only a limited overall health benefits effect in the subjects in the present cohort, who had relatively high baseline levels of *Akkermansia* spp. in spite of their metabolic deviations.

The safety and tolerability profile of pasteurized *A. muciniphila* MucT was excellent and consistent with previous observations in studies in which both live and pasteurized *A. muciniphila* MucT^9^ ^,^ ^10^ The number of adverse events was well balanced between the pasteurized *A. muciniphila* MucT and placebo arm. Interestingly, a relatively lower number of participants with infections were observed in the arm that was treated with pasteurized *A. muciniphila* MucT compared to placebo.

Beyond the results already highlighted, several additional considerations help contextualize the metabolic effects observed with pasteurized *A. muciniphila* MucT. First, the 16-week intervention period may have been insufficient to capture the full metabolic impact of the supplementation. Improvements in insulin sensitivity, particularly in individuals with long-standing metabolic syndrome, often require longer exposure, which may explain why we observed different responses at three versus four months. Based on the observed differences in HOMA-IR and Matsuda values, we also propose that hepatic insulin sensitivity is improved faster than whole-body insulin sensitivity as a result of the administration of pasteurized *A. muciniphila* MucT. Second, this cohort displayed unexpectedly high relative baseline levels of *Akkermansia* spp., in some cases exceeding those of geographically matched healthy controls. This unusual feature likely limited the room for improvement in the overall analysis and emphasizes the relevance of stratifying participants by low baseline relative abundance of *Akkermansia* spp. when this was achieved, the strongest effects were indeed detected. Third, the absence of broad changes in gut microbiota composition should not be viewed as a limitation. Rather, it aligns with the expected behavior of a pasteurized strain that cannot colonize the colon and likely already acts in the upper intestinal tract. Finally, two additional findings add biological plausibility to the observed benefits in the low-*Akkermansia* subgroup. The improvement in renal function echoes preclinical evidence linking gut barrier dysfunction to kidney impairment. Likewise, the reduction in android and trunk fat mass supports the existence of a “gut barrier‒visceral adipose tissue axis,” a key driver of insulin resistance.^37^

This study has several strengths and weaknesses. A perceived weakness is the heterogeneity in terms of the inclusion of both normo- and dysglycemic participants due to recruitment issues during the COVID-19 pandemic. Moreover, the number of post-OGTT assays was necessarily limited as only small amounts of blood could be collected. As there was a negative primary study outcome, all subsequent analyses were exploratory and needed to be interpreted accordingly. A major strength, though, is the randomized, double-blind, placebo-controlled trial design allowing to test the impact of the pasteurized *A. muciniphila* MucT intervention for a 16-week period with detailed assessments of body composition and biochemical parameters at fasting and after an oral glucose tolerance test. Moreover, deep metagenomic analysis of the gut microbiota allowed for species level and richness analysis.

In conclusion, this is the first well-controlled study addressing the impact of pasteurized *A. muciniphila* MucT in subjects with metabolic syndrome and obesity. Despite the lack of effects on whole-body insulin sensitivity in the overall study population, highly significant and clinically relevant cardiometabolic improvements were observed in subjects with low levels of *Akkermansia* spp. that are globally prevalent. Because the European Food Safety Authority^38^ has determined that only pasteurized *A. muciniphila* MucT is suitable for human consumption, this finding supports the use of this specific supplement in individuals who may benefit from it. While the global effects were modest, the study strongly suggests that individuals with low baseline *Akkermansia* levels derive meaningful and clinically relevant metabolic and anthropometric benefits, reinforcing the potential for microbiome-informed personalized nutrition.

## Supplementary Material

Supplementary material Supplementary Materials Responder Analysis.xlsx

Supplementary Materials Differential abundance.xlsx

Supplementary material Supplementary Figures

## Data Availability

The metagenome sequences in this study have been deposited in the European Nucleotide Archive (ENA) under accession code PRJEB106335.
