# Supplementary material for: Effect of pasteurized Akkermansia muciniphila MucT on insulin sensitivity, body composition, and GLP-1 production in subjects with metabolic syndrome: impact of low baseline gut Akkermansia levels
Source: Gut Microbes. 2026 Jun 24;18(1):2690689. doi: 10.1080/19490976.2026.2690689 (PMC13313273; doi:10.1080/19490976.2026.2690689)
Supplement: Supplementary material — Supplementary Figures [file KGMI_A_2690689_SM5571.docx]

**SUPPLEMENTARY FIGURES**


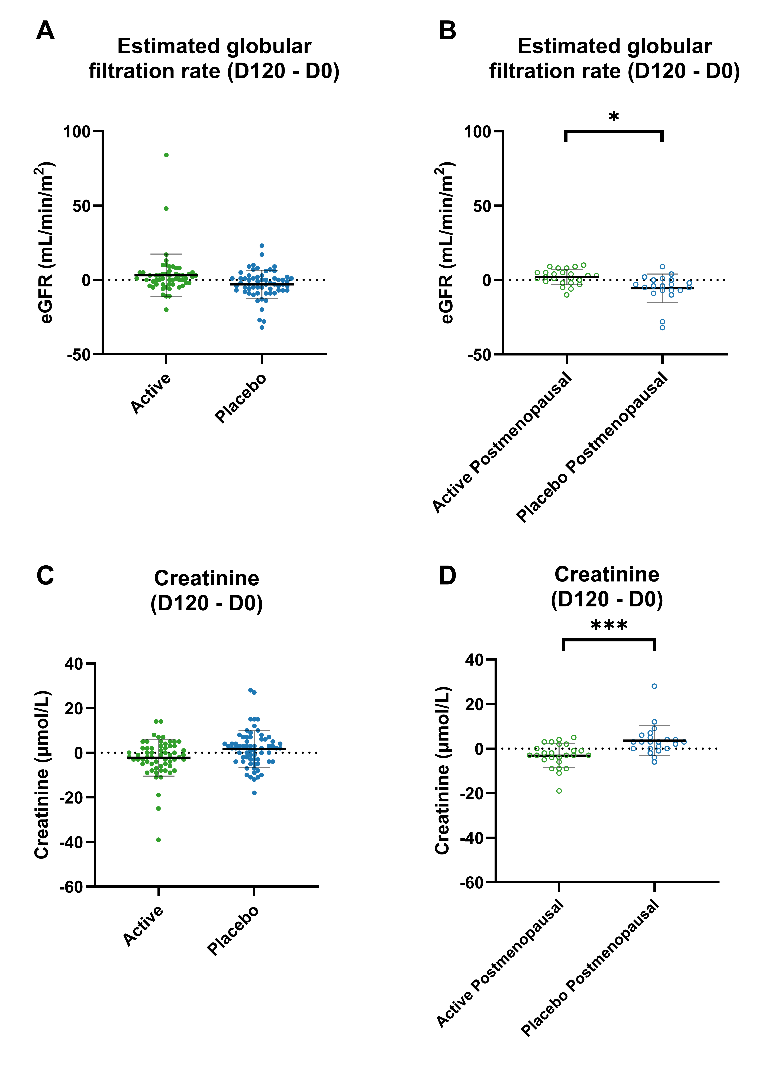


Supplementary Figure S1: Figure 3: A, B. Estimated globular filtration rate and C, D. Creatinine changes after 4 (D120) months of treatment in all subjects (A, C) and postmenopausal women (B, D). *: p<0.05, ***: <0.001.


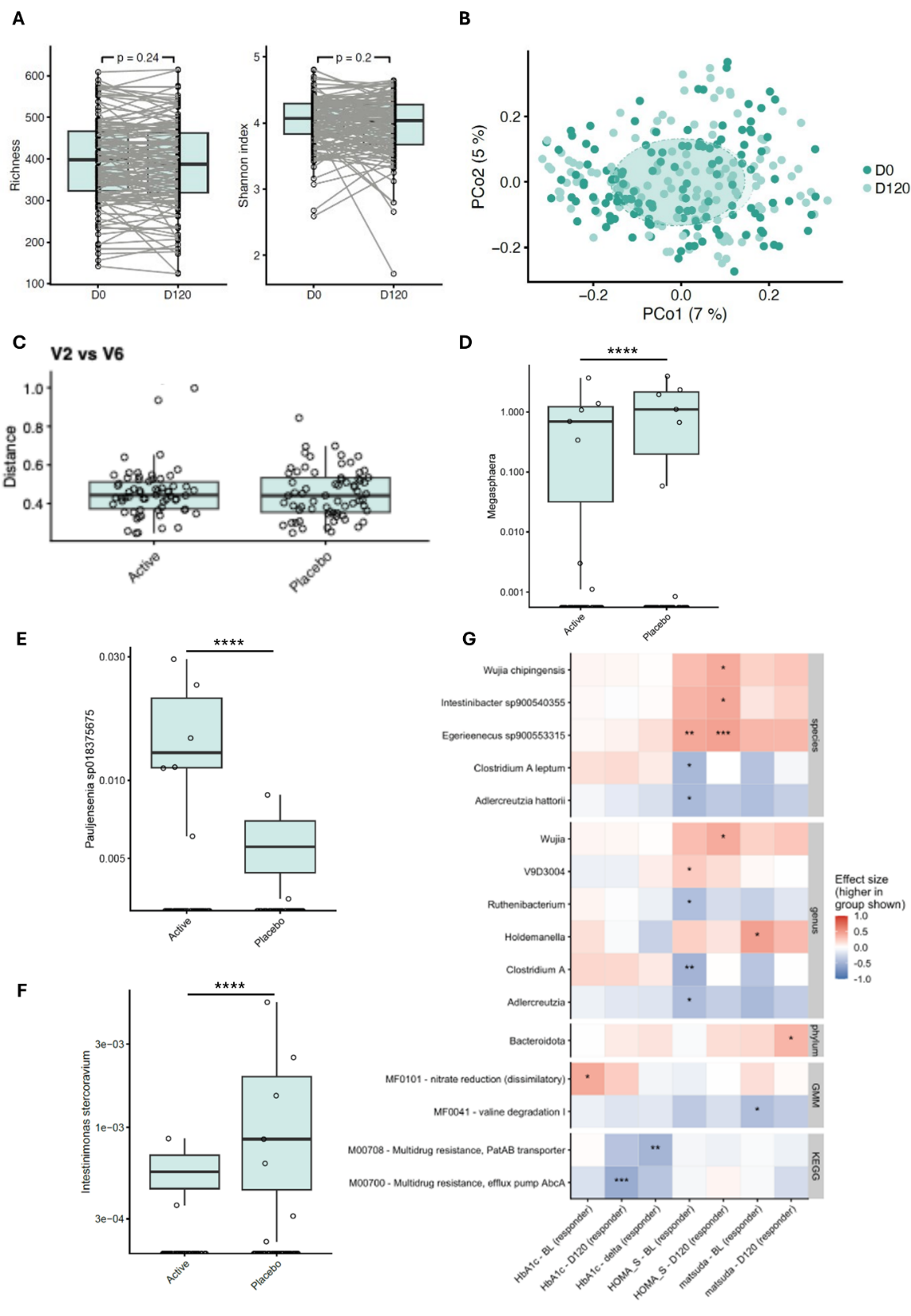


Supplementary Figure S2. A. Boxplots showing differences in species richness and Shannon diversity for all subjects. Paired statistical tests were done using a Wilcoxon signed rank test. B . Principal Coordinate Analysis of Bray-Curtis dissimilarity of all samples colored by visit. C. Boxplots showing differences in Bray-Curtis dissimilarity between D0 and D120 in active and placebo group. D-F. Relative abundance of taxa that showed significant differences in delta D120-D0 (D) or at D120 (E, F) between active and placebo groups. G. Heatmap showing the significant results of responder vs. non-responder analysis. Groups were compared using Wilcoxon signed-rank test. Significance is defined by FDR-corrected p-values as* < 0.05, ** < 0.01, *** < 0.001, **** < 0.0001. BL: Baseline; D: Day; HbA1c: Hemoglobin A1C; HOMA_S: Homeostatic Model Assessment for Insulin Sensitivity, Pco: Principal coordinate.


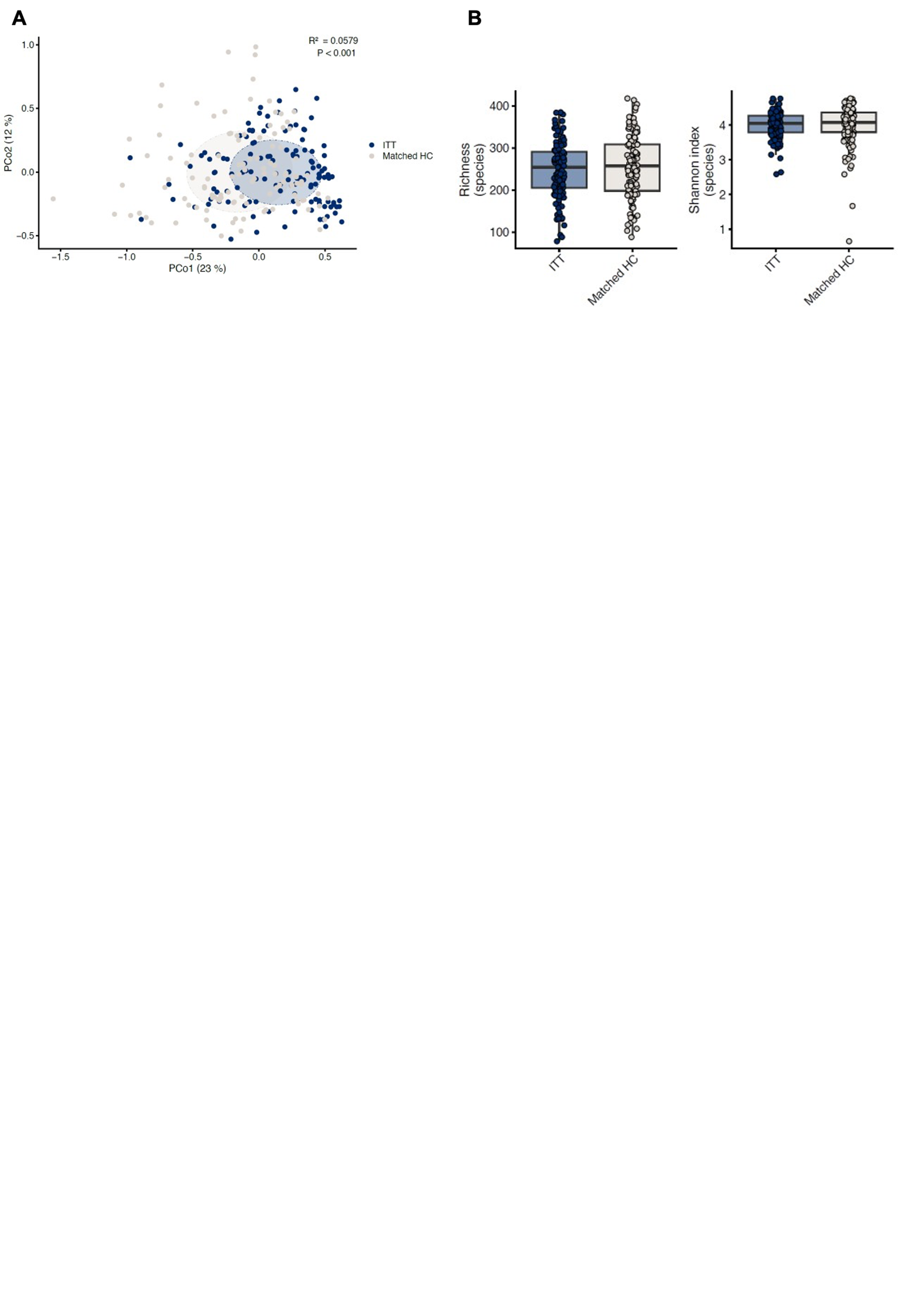


Supplementary Figure S3. A. Principal Coordinate Analysis showing separation of ITT subjects at baseline and their matched healthy controls. B Richness and Shannon index of ITT subjects at baseline and their matched healthy controls. HC: healthy controls, PCo: principal coordinate, ITT: intention-to-treat.


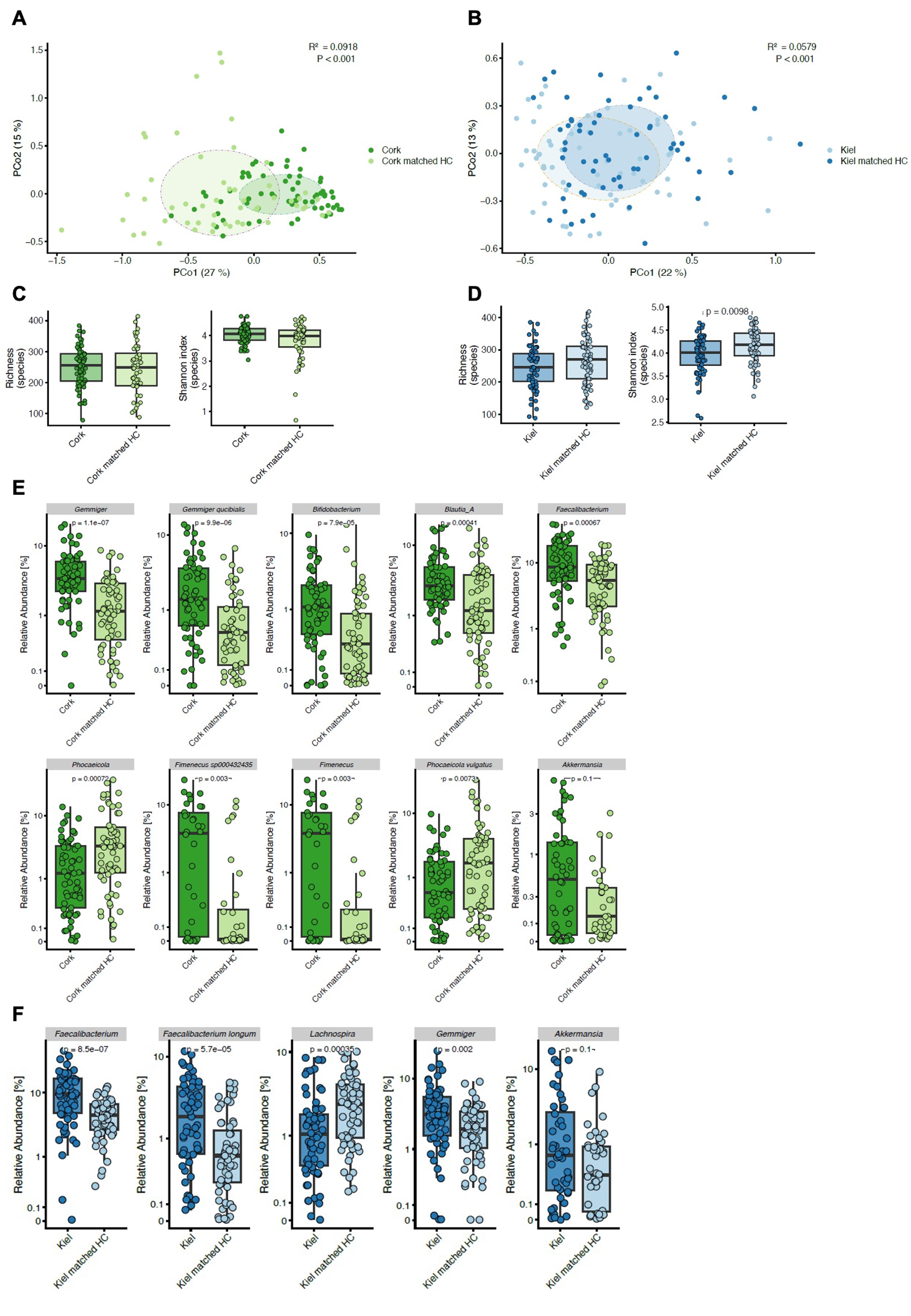


Supplementary Figure S4. A, B. Principal Coordinate Analysis showing separation of ITT subjects at baseline and their matched healthy controls for Cork and Kiel, respectively. C, D. Richness and Shannon index of ITT subjects at baseline and their matched healthy controls for Cork and Kiel, respectively. E, F. Relative abundance of taxa that show significant differences and Akkermansia between ITT and their matched healthy controls for Cork and Kiel, respectively. PCo: principal coordinate, ITT: intention-to-treat.

**SUPPLEMENTARY TABLES**

Supplementary Table S1. Inclusion and exclusion criteria.

| **Inclusion criteria** | **Exclusion critera** |
| --- | --- |
| - Give written informed consent; - Males & females aged between 21 to 75 years, inclusive; - Have a BMI between >25 and <40 Kg/m^2^; - Qualifying for the diagnosis of metabolic syndrome according to the International Diabetes Federation (IDF 2006) criteria. At least any three of five criteria, with the modification that the criterion FPG≥100 mg/dL (5.6 mmol/L) is required among at least three of five:   - Increased waist circumference: for Europid, sub-Saharan African, Eastern and Middle-Eastern ≥94 cm (men) or ≥80 cm (women), with ethnic-specific waist circumference cut-points: for South Asian and Chinese patients, waist ≥90 cm (men) or ≥80 cm (women); for Japanese patients, waist ≥90 cm (men) or ≥80 cm (women);   - Triglycerides ≥150 mg/dL (1.7 mmol/L) (*exception:* triglycerides ≥100 mg/dL (1.13mmol/L) for sub-Saharan African) or treatment for elevated triglycerides;   - HDL cholesterol <40 mg/dL (1.03 mmol/L) in men or <50 mg/dL (1.29 mmol/L) in females, or treatment for low HDL;   - Systolic blood pressure ≥130, diastolic blood pressure ≥85 mmHg, or treatment for hypertension;   - FPG ≥100 mg/dL (5.6 mmol/L) or previously diagnosed type 2 diabetes; an oral glucose tolerance test is recommended for patients with an elevated FPG, but it is not required. Having an FPG ≥100 mg/dL (5.6 mmol/L) is required. - If participant has a prior diagnosis of pre-diabetes or Type II diabetes, he/she must have been unmedicated for 3-months prior to screening; - If female, must meet all the following criteria:   - Not pregnant or breastfeeding   - If of childbearing potential (including peri-menopausal women who have had a menstrual period within one year) must practice and be willing to continue to practice appropriate birth during the entire duration of the study - Be willing to maintain stable dietary habits and physical activity levels throughout the trial period; - Be able to communicate well with the Investigator, to understand and comply with the requirements of the study and be judged suitable for the study in the opinion of the Investigator. | - Uncontrolled hyperglycemia assessed by HbA1c ≥ 6.5%; - Suffering from a metabolic disorder such as diabetes mellitus that requires lifestyle and dietary recommendations or a medication according to the recommendations, uncontrolled thyroidal trouble (Confirmed by clinical significant abnormal TSG/T4 and/or stabled medication for >3 months) or other metabolic disorder; - Suffering from a severe chronic disease (e.g. cancer, HIV, renal failure, hepatic or biliary disorders ongoing, chronic inflammatory digestive disease, inflammatory bowel disease, irritable bowel syndrome, arthritis or other chronic respiratory trouble, etc.) or gastrointestinal disorders found to be inconsistent with the conduct of the study by the investigator (e.g. celiac disease); - With a history of retinopathy, microalbuminuria, ischemic cardiovascular event during the previous 6 months; - Consumption of more than 30 g of dietary fibre per day, as measured by the Block Fibre Screener - Prior diagnosis of Type I diabetes mellitus (i.e. a clinical diagnosis made before the screening visit of this study); - Alcohol consumption (>21 units per week); - Smoking more than 10 cigarettes per day; - Previous bariatric surgery; - Any surgery in the 3 months before the study or planned for 6 months after enrolling; - Pregnancy or pregnancy planned in the 6 months after enrolling or lactating women; - Consumption of dietary supplements (omega-3 fatty acids, probiotics, prebiotics, plant stanols/sterols) in the 4-weeks before the study; - Presence or history of significant and diagnosed gastrointestinal diseases that, in the opinion of the investigator, could be associated with disturbed gastrointestinal absorption (e.g. resections, diverticula, active and diagnostically confirmed irritable bowel syndrome, malabsorption syndrome); - Present or recent (within 3-months of screening) use of any other medication which, in the opinion of the investigator, could interfere with the outcome of the study, including but not limited to antithrombotic agents, anti-inflammatory agents and chronic NSAID use (except low-dose prophylactic, proton pump inhibitors (PPIs), antihistamines, if ongoing (3-months) and on a stable dose throughout study period); - Steroids (over the counter (OTC) NSAIDS, topical steroids and inhalers are allowed) - Current or planned participation in a weight-loss regimen (including intermediate fasting), including extreme dietary practices or exercise; - Anorexia nervosa, bulimia or significant eating disorders according to the investigators; - Having lost >5% of their body weight within 3-months prior to screening; - Lactose intolerance or milk protein allergy; - Gluten intolerance; - Current treatment with medications influencing the parameters of interest (glucose-lowering drugs such as metformin, DPP4 inhibitors, GLP-1 receptor agonists, acarbose, sulfonylureas, glinides, thiazolidinediones, sodium-glucose cotransporter -2 inhibitors, insulin, lactulose, glucocorticoids, immunosuppressive agents); - Antibiotic use in the 3 months before the study; - Participant has a known allergy to inactive or active ingredients in the study products; - Participation in other clinical research trials within 30 days prior to randomization; - Any other condition which in the Investigator’s opinion may adversely affect the subject’s ability to complete the study or its measures or which may pose significant risk to the subject. |

Supplementary Table S2. Estimated Differences by pasteurized *A. muciniphila* vs. Placebo in insulin-sensitivity-related endpoints in ITT population and prediabetes subpopulation in function of low fecal Akkermansia muciniphila MucT level at baseline, postmenopausal status and age.

| ***Endpoint*** | **D0 - D90** | ***p-value*** | **D0 - D120** | ***p-value*** |
| --- | --- | --- | --- | --- |
| ***MATSUDA index* primary*** | 1.052 (0.917 to 1.208) | *0.463* | 0.958 (0.829 to 1.107) | *0.557* |
| *Low Akk* | 1.279 (1.075 to 1.521) | ***0.022*** | 1.126 (0.934 to 1.357) | *0.291* |
| *Postmenopause* | 1.17 (0.942 to 1.464) | *0.146* | 1.219 (0.989 to 1.504) | *0.065* |
| *63y or Older* | 1.097 (0.891 to 1.351) | *0.374* | 1.074 (0.881 to 1.308) | *0.473* |
| ***MATSUDA index* ITT*** | 1.066 (0.948 to 1.199) | *0.282* | 1.020 (0.907 to 1.148) | *0.737* |
| *Low Akk* | 1.263 (1.068 to 1.493) | ***0.007*** | 1.161 (0.976 to 1.381) | *0.09* |
| *Postmenopause* | 1.17 (0.945 to 1.447) | *0.145* | 1.207 (1.018 to 1.432) | ***0.031*** |
| *63y or Older* | 1.138 (0.935 to 1.384) | *0.192* | 1.13 (0.952 to 1.341) | *0.159* |
| **HOMA-S (%) *primary*** | 12.07 (-0.223 to 24.369) | *0.054* | 2.13 (-12.687 to 16.955) | *0.776* |
| Low Akk | 33.038 (12.40 to 53.677) | ***0.002*** | 20.592 (-3.933 to 45.117) | *0.097* |
| *Postmenopause* | 9.8 (-6.381 to 26.007) | *0.312* | 4.731 (-10.125 to 19.588) | *0.593* |
| *63y or Older* | 16.287 (2.619 to 29.956) | *0.051* | 9.058 (-9.198 to 27.314) | *0.41* |
| ***HOMA-S (%) ITT*** | 8.912 (-0.834 to 18.657) | *0.073* | 1.467 (-10.573 to 13.511) | *0.810* |
| Low Akk | 25.841 (10.50 to 41.181) | ***0.001*** | 13.322 (-5.620 to 32.264) | *0.165* |
| *Postmenopause* | 11.635 (-1.394 to 24.664) | *0.141* | 4.129 (-8.829 to 17.088) | *0.595* |
| *63y or Older* | 14.065 (-0.102 to 28.231) | *0.052* | 7.934 (-11.750 to 27.617) | *0.423* |
| ***HOMA-IR ITT*** | -0.244 (-0.731 to 0.243) | *0.323* | -0.150 (-0.619 to 0.318) | *0.425* |
| Low Akk | -0.63 (-1.331 to 0.072) | *0.077* | -0.445 (-1.171 to 0.28) | *0.224* |
| *Postmenopause* | -0.595 (-1.435 to 0.245) | *0.238* | -0.852 (-1.639 to -0.065) | *0.076* |
| *63y or Older* | -0.298 (-1.118 to 0.522) | *0.546* | 0.053 (-0.677 to 0.783) | *0.904* |

*Data are expressed as estimated means (95% confidence intervals). Mean changes were estimated from a mixed model for repeated measures with time, product and time-product interactions as variables adjusting for the baseline parameter measured, metabolic syndrome criteria and site. *Geometric mean: data are transformed using log10 and express a mean ratio. HbA1c: Hemoglobin A1C, HDL: High-density lipoprotein, HOMA-IR: Homeostatic Model Assessment for Insulin Resistance, HOMA-S: Homeostatic Model Assessment for Insulin Sensitivity, ITT: Intention to treat, LDL: Low-density lipoprotein.*

Supplementary Table S3. Difference between active and placebo in GLP-1 response to oral glucose tolerance test.

| **Group** | **Visit day** | **Geometric Mean** | **95% Lower CL** | **95% Upper CL** | **P-Value** |
| --- | --- | --- | --- | --- | --- |
| **ITT** | 90 | 1.1916 | 1.0688 | 1.3285 | **0.002** |
| **ITT** | 120 | 1.0778 | 0.96266 | 1.2069 | 0.192 |
| **High baseline** | 90 | 1.2190 | 1.0257 | 1.4487 | **0.025** |
| **High baseline** | 120 | 1.0179 | 0.8692 | 1.1921 | 0.823 |
| **Low baseline** | 90 | 1.1925 | 1.0245 | 1.3881 | **0.024** |
| **Low baseline** | 120 | 1.1771 | 0.9840 | 1.4080 | 0.074 |

Supplementary Table S4. Baseline characteristics of low and high baseline level subgroups

| **Characteristics ITT population** | ***A. muciniphila,* low baseline, n=29** | ***A. muciniphila,* high baseline, n=32** | **Placebo, low baseline, n=35** | **Placebo, high baseline, n=32** |
| --- | --- | --- | --- | --- |
| **Site: Cork / Kiel** | 16 / 13 | 15 / 17 | 18 / 17 | 16 / 16 |
| **Age (years)** | 61 ± 9 | 64 ± 10 | 57 ± 11 | 62 ± 9 |
| **Women (no (%))** | 15 (52%) | 17 (53%) | 21 (60%) | 19 (59%) |
| **Postmenopausal women (no (%))** | 11 (38%) | 12 (38%) | 10 (29%) | 11 (34%) |
| **Body weight (kg)** | 93.88 ± 15.76 | 95.80 ± 13.12 | 92.90 ± 13.27 | 92.78 ± 15.79 |
| **BMI (kg/m^2^)** | 32.05 ± 3.32 | 32.83 ± 4.40 | 32.34 ± 3.72 | 31.14 ± 3.21 |
| **Body fat percentage (%)** | 38.04 ± 6.43 | 39.63 ± 8.59 | 38.38 ± 7.55 | 37.67 ± 7.28 |
| **Total body fat mass (kg)** | 35.42 ± 7.24 | 38.20 ± 10.93 | 35.56 ± 8.58 | 34.97 ± 8.67 |
| **Total body lean mass (kg)** | 58.46 ± 12.45 | 57.69 ± 9.99 | 57.27 ± 11.57 | 57.44 ± 12.38 |
| **Waist circumference (cm)** | 105.51 ± 11.42 | 107.55 ± 11.22 | 104.09 ± 10.84 | 104.44 ± 12.37 |
| **Hip circumference (cm)** | 111.11 ± 8.75 | 113.14 ± 9.82 | 110.18 ± 7.80 | 110.94 ± 7.82 |
| **Matsuda index** | 3.57 ± 2.43 | 3.57 ± 1.71 | 3.24 ± 1.77 | 3.61 ± 2.09 |
| **HOMA-S%** | 71.20 ± 36.95 | 73.41 ± 32.96 | 73.44 ± 35.36 | 79.58 ± 33.92 |
| **HOMA-IR** | 3.56 ± 2.31 | 3.34 ± 1.86 | 3.62 ± 1.78 | 2.80 ± 1.45 |
| **FBG ≥ 5.6 mmol/L (no (%))** | 27 (96%) | 29 (91%) | 28 (80%) | 27 (84%) |
| **HbA1c (mmol/mol)** | 37.45 ± 4.04 | 36.26 ± 3.79 | 35.69 ± 4.13 | 35.91 ± 3.62 |
| **Total cholesterol (mmol/L)** | 5.16 ± 1.27 | 5.03 ± 1.17 | 5.40 ± 1.09 | 5.27 ± 1.08 |
| **HDL-Cholesterol (mmol/L)** | 1.33 ± 0.33 | 1.28 ± 0.28 | 1.33 ± 0.33 | 1.34 ± 0.18 |
| **LDL-Cholesterol (mmol/L)** | 3.28 ± 1.31 | 3.34 ± 1.06 | 3.61 ± 0.99 | 3.47 ± 1.09 |
| **Triglycerides (mmol/L)** | 1.51 ± 0.77 | 1.43 ± 0.72 | 1.77 ± 1.02 | 1.50 ± 0.58 |

*Data expressed as observed means (standard deviation). No statistical difference was found between the two groups. BMI: Body mass index, HbA1c: Hemoglobin A1C, HDL: High-density lipoprotein, HOMA-IR: Homeostatic Model Assessment for Insulin Resistance, HOMA-S: Homeostatic Model Assessment for Insulin Sensitivity, ITT: Intention to treat, LDL: Low-density lipoprotein, no: Number*

Supplementary Table S5. Estimated Differences by pasteurized *A. muciniphila* vs. Placebo in metabolic health-related endpoints in ITT low fecal *Akkermansia* level at baseline.

| ***Endpoint*** | **D0 - D90** | ***p-value*** | **D0 - D120** | ***p-value*** |
| --- | --- | --- | --- | --- |
| ***Fasting Plasma Glucose (mmol/L) ITT*** | 0.001 (-0.143 to 0.144) | *0.991* | 0.084 (-0.109 to 0.276) | *0.391* |
| Low Akk | -0.017 (-0.209 to 0.175) | *0.86* | 0.0638 (-0.181 to 0.309) | *0.605* |
| ***HbA1c (mmol/mol) ITT*** | -0.304 (-1.19 to 0.582) | *0.499* | -0.28 (-1.383 to 0.823) | *0.612* |
| Low Akk | -0.537 (-1.768 to 0.695) | *0.387* | -0.739 (-2.205 to 0.727) | *0.317* |
| ***Postprandial glycemia (3-hour OGTT) ITT*** | 0.182 (-0.182 to 0.547) | *0.324* | 0.244 (-0.154 to 0.642) | *0.228* |
| Low Akk | 0.0168 (-0.486 to 0.52) | *0.947* | -0.061 (-0.682 to 0.56) | *0.845* |
| ***Postprandial insulinemia (3-hour OGTT) ITT*** | -2.906 (-10.546 to 4.735) | *0.453* | -2.914 (-10.849 to 5.022) | *0.469* |
| Low Akk | -8.862 (-18.01 to 0.286) | *0.057* | -7.506 (-18.605 to 3.593) | *0.181* |
| ***Postprandial triglyceridemia (3-hour OGTT) ITT*** | 0.161 (-0.138 to 0.460) | *0.289* | 0.140 (-0.056 to 0.336) | *0.160* |
| Low Akk | -0.051 (-0.312 to 0.21) | *0.695* | 0.194 (-0.138 to 0.526) | *0.247* |
| ***Total cholesterolemia (mmol/L) ITT*** | -0.023 (-0.320 to 0.273) | *0.876* | 0.072 (-0.150 to 0.294) | *0.523* |
| Low Akk | 0.083 (-0.334 to 0.500) | *0.692* | 0.138 (-0.229 to 0.506) | *0.454* |
| ***HDL-cholesterolemia (mmol/L) ITT*** | -0.060 (-0.119 to -0.002) | ***0.043*** | 0.028 (-0.027 to 0.083) | *0.318* |
| Low Akk | -0.036 (-0.119 to 0.047) | *0.389* | 0.025 (-0.059 to 0.108) | *0.553* |
| ***LDL-cholesterolemia (mmol/L) ITT*** | 0.04 (-0.191 to 0.271) | *0.735* | 0.041 (-0.161 to 0.243) | *0.689* |
| Low Akk | 0.172 (-0.198 to 0.54) | *0.356* | 0.076 (-0.225 to 0.378) | *0.614* |
| ***Triglyceridemia (mmol/L) ITT*** | 0.157 (-0.121 to 0.434) | *0.352* | 0.133 (-0.053 to 0.320) | *0.239* |
| Low Akk | 0.01 (-0.265 to 0.285) | *0.942* | 0.266 (-0.16 to 0.691) | *0.216* |

*Data are expressed as estimated means (95% confidence intervals). Mean changes were estimated from a mixed model for repeated measures with time, product and time-product interactions as variables adjusting for the baseline parameter measured, metabolic syndrome criteria and site. HbA1c: Hemoglobin A1C, HDL: High-density lipoprotein, ITT: Intention to treat, LDL: Low-density lipoprotein, OGTT: oral glucose tolerance test.*

Supplementary Table S6. Summary of most commonly occurring adverse events (N=142)

| **Event** | **All participants (n=142)** | **Placebo (n=72)** | ***A.* *muciniphila* (n=70)** |
| --- | --- | --- | --- |
| *number of participants (%)* | | | |
| Any AE | 104 (73%) | 53 (74%) | 51 (73%) |
| Serious AE |  |  |  |
| pneumonia | 1 (0,7%) | 1 (1,4%) | 0 |
| AEs that led to discontinuation of treatment or placebo | 2 (1,4%) | 0 | 2 (3%) |
| AEs that led to early withdrawal from the trial | 0 | 0 | 2 (3%) |
| AEs reported by PI as unlikely to probably related | 56 (39%) | 25 (35%) | 31 (44%) |
| All AEs that occurred in at least 5% of all participants |  |  |  |
| Corona virus infection | 20 (14%) | 13 (18%) | 7 (10%) |
| Nasopharyngitis | 20 (14%) | 13 (18%) | 7 (10%) |
| Abdominal discomfort | 15 (11%) | 6 (8%) | 9 (13%) |
| Hypertension or blood pressure increased | 12 (9%) | 3 (4%) | 9 (13%) |
| Diarrhoea | 8 (6%) | 2 (3%) | 6 (9%) |
| Flatulence | 8 (6%) | 3 (4%) | 5 (7%) |
| Influenza like illness | 8 (6%) | 3 (4%) | 5 (7%) |
| Liver enzymes increased | 8 (6%) | 2 (3%) | 6 (9%) |
| Infections |  |  |  |
| All infections | 60 (42%) | 35 (49%) | 25 (36%) |
| Respiratory infections | 41 (29%) | 25 (35%) | 16 (23%) |
| Gastrointestinal infections | 10 (7%) | 5 (7%) | 5 (7%) |
| Inflammatory parameter: |  |  |  |
| CRP increased | 7 (5%) | 5 (7%) | 2 (3%) |
| Number (percent) of Participants with Adverse Events (AE) and Serious Adverse Events after Randomization | | | |
